# Energy saving for OpenFlow switch on the NetFPGA platform based on queue engineering

**DOI:** 10.1186/s40064-014-0775-8

**Published:** 2015-02-06

**Authors:** Tran Hoang Vu, Vu Cong Luc, Nguyen Trung Quan, Nguyen Huu Thanh, Pham Ngoc Nam

**Affiliations:** Hanoi University of Science and Technology, Hanoi, Vietnam

**Keywords:** OpenFlow switch, NetFPGA, Low power, Data center network, Green networking

## Abstract

Data centers play an important role in our daily activities. The increasing demand on data centers in both scale and size has led to huge energy consumption that rises the cost of data centers. Besides, environmental impacts also increase considerably due to a large amount of carbon emissions. In this paper, we present a design aimed at *green networking* by reducing the power consumption for routers and switches. Firstly, we design the *Balance Switch* on the NetFPGA platform to save consumed energy based on Queue Engineering. Secondly, we design the test-bed system to precisely measure the consumed energy of our switches. Experimental results show that energy saving of our switches is about *30% - 35%* of power consumption according to variation of input traffic compared with normal Openflow Switch. Finally, we describe performance evaluations.

## Introduction

One of the most important issues that many researches of today society are very concerned for is to save energy consumption in data centers. A research, the Datacenter Dynatmics 2012 Global census shown that the consumed power in data centers between 2011 and 2012 rose significantly to 63% with 38GW (Sverdlik et al. 2011); in which the network devices consumes around from 20% to 30% of this energy (Heller et al. 2010; The Green Grid). On the other hand, the cost of consumed energy on data centers was about 44% of total costs (U.S. Environmental Protection Agency’s Data Center Report to Congress). At the same time, along with huge energy consumption, data centers also emitted a large amount of carbon dioxide.

**Figure 1:**
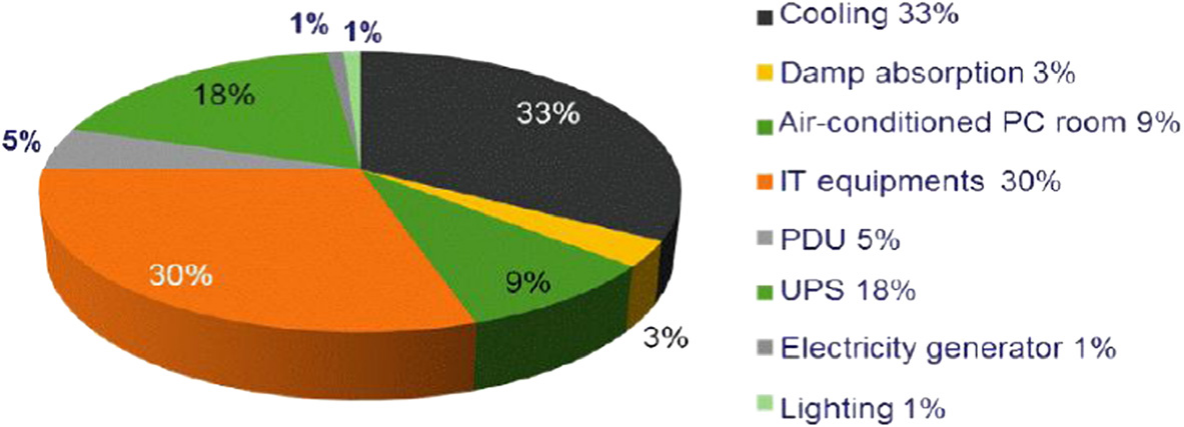
**Energy consumption in data center** **(** The Green Grid **).**

In (The Green Grid), we can also see that energy consumption of components in a data center (Figure 1). It is clear that the proportion of the energy consumption of IT devices in the data center is significant, about 30%. Thus, one of the most controversial issues today relates to saving the energy consumption of network devices.

**Figure 2:**
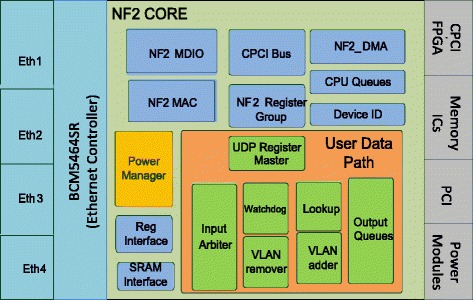
**Design of Power Manager Block for the Openflow Switch.**

There are some solutions to solve this problem such as reducing the clock rate from125 MHz to 62.5 MHz by changing the value of a hardware register according to the input bit rate (Lombardo et al. 2012). In this solution, the frequency is only reduced to a half of the original frequency and this clock frequency variation only affects a part of the NetFPGA board. Another method proposed in (Meng et al. 2012), the clock frequency is divided into lower levels from 125 MHz to 3.096MH according to the real time workload. However, it only applies to pipeline blocks in the User Data Path (UDP) (Figure 2). In addition, in (Hanay et al. 2012) given another approach that changes the link rate on the ports (1GB/100Mb/10 Mb) according to the queue length by reducing the frequency of Ethernet MAC block to 25 MHz. In this method, the research is only applied for particular Ethernet MAC blocks. Overall, these solutions are not really effective in saving the consumed energy because of some above- analyzed restrictions.

Moreover, in (Thanh et al. 2012), (Heller) shown that the traffic through switches and routers varies significantly according to time the input traffic peaks during the day and falls at night. However, the energy consumption of these devices remains constant that wastes energy. Thus, we should design the new network devices that consume energy according to the input traffic. In this paper, we describe a design – ***Balance Switch*** to save the power consumption of OpenFlow switches using in data centers by changing the operating frequence according to the input traffic. The main contributions of our work are as follows:

- We design a new module - Power Manager (PM) for Openflow Switch. This function can manage and automatically control to change operating modes of switches including ***IDLE mode, WORKING mode*** and ***SLEEP mode.*** So our method does not drop packets compared with these methods in (Lombardo et al. 2012) and (Meng et al. 2012) that can drop packets.
- We also design a new module - Clock Controller (CC) to control the frequence of switches. Based on the control signals of the PM block, at ***WORKING mode*** and ***IDLE mode***, CC maintains the operating frequence at 125 MHz; whereas the frequence is reduced to 0 MHz at ***SLEEP mode*** in order to save the consumed energy of Openflow Switch.
- We build a test-bed system and precisely measure the consumed energy of the Openflow Switch, based on PCIEXT-64UB kit (Product Specifications and User Manual of PCI-EXT64U/UB and PCIeEXT-16HOT). Experimental results show that energy saving is around ***30-35%.***

The rest of this paper is organized into following main parts: Section II – Design of Power Manager Block for the OpenFlow Switch. Section III – Design of Clock Controller Block for the Openflow Switch. Section IV – Description of Experimental Results. Section V - Description of Evaluation Testbed System. Finally, section VI - Conclusions.

## Design of power manager block for the openflow switch

### The method to change the frequence based on queue engineering

Generally, a network device that saves consumed energy operates in two modes: working mode and sleep mode. When the device is actively processing traffic, it is operating in the working mode. When there is no traffic for processing, the device is changed to the sleep mode to save energy. Thus, the energy consumption of a general network device could be modeled as:

1 $$ \mathrm{E} = {\mathrm{P}}_{\mathrm{working}}*\ {\mathrm{T}}_{\mathrm{working}} + {\mathrm{P}}_{\mathrm{sleep}}*{\mathrm{T}}_{\mathrm{sleep}} $$

Where T_working_ and T_sleep_ denote the time spent in the working mode and sleep mode, respectively. P_working_ and P_sleep_ represent the power consumption in each mode.

In a published paper (Anderson et al. 2004), the power consumption of a CMOS device, which is the FPGA chip in our circumstance, is in proportional to the operating clock frequency ans can be calculated with the equation below:

2 $$ {\mathrm{P}}_{\mathrm{avg}}=\frac{1}{2}{\displaystyle \sum_{n\mathit{\in} nets}{C}_n}.{f}_n.{V}^2 $$

**Figure 3:**
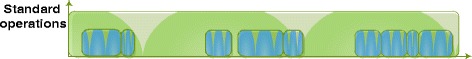
**Activity of Normal switch.**

**Figure 4:**
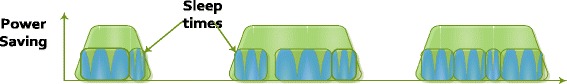
**Activity of power saving switch.**

Where P_avg_ represents the average power consumption, *C*_n_ is the capacitance of a net n, V is the voltage supply, and *f*_*n*_ is the average toggle rate (switching activity) of net n. According to the formula (2), we can easily see how the power interrelates with the operating frequency. In order to save the energy consumption, we proposed that the switch should be sleep by reducing the operating frequency to 0 MHz when there is no traffic flowing through the switch (Figure 3 and Figure 4).

**Figure 5:**
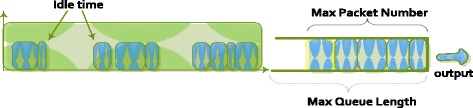
**Activity of Balance Switch.**

However, when a packet is sent to an Openflow Switch, the switch receives completely this packet on the input queue, then it starts processing and forwarding to other network devices. With the aim to increase energy saving for the Openflow Switch, we also raise T_sleep_. Packets contain on input queue and wait for a threshold, then the switch is waked up to process and forward these packets (Figure 5).

In our method, when the queue length or the number of packets at the input queue or wait time of a first packet equals to their thresholds (Max Queue Length, Max Packet Number, Wait Timeout, respectively), the switch changes to its normal state with operating frequency of 125 MHz.

### Describe the power manager block on the openflow switch

We would like to analyze contributions of different functional blocks embedded in the FPGA chip on the Openflow Switch (Figure 2) (Naous et al. 2008a) (OpenFlow Switch Specification Version 1.1.0 Implemented (Wire Protocol 0x02)). Speaking to the OpenFlow Switch designed in FPGA, *NF2_TOP* is the top level of switch architecture, where all entities are described and linked to each other. It is highlighted that the main functional block in this design is *NF2_CORE*; the outside of this block only contains digital clock manager units while the inside has the following implemented functional blocks: CPCI Bus, NF2 DMA, CPU Queue, NF2 Reg grp, User Data Path, NF2 MDIO, and NF2 Mac etc. These blocks are connected to four Ethernet ports through one NIC of Broadcom BCM5464SR that controls the operation of these ports. Besides, the Openflow Switch also includes some other components such as the power modules, memory device, PCI link, CPCI Bridge chip, and so forth.

In order to detect automatically the packets that are sent to the switch and manage states of the switch such as working or idle, we have designed the Power Manager block (Figure 2). This block is built and functions to summarize the input signals from other blocks on NF2 Core. It provides an output signal to control the operating frequency of switch. The function of some main blocks on NF2 Core is described below:

- CPCI Bus block connects to CPCI Chip (Chip spartan 2) ((2014) “NetFPGA-1G-CML™ Board Reference Manual” Revised January 28 2014) by Bus CPI in order that PC can communicate with registers on the Openflow Switch.
- NF2 DMA block also connects to CPCI Chip by Bus DMA in order to transmit data to and receive them (packets through Ethernet/IP) from CPU Queue.
- CPU Queue: This block includes CPU RX Queue and CPU TX Queue. Packets shall be transferred from the CPU RX queue in the NetFPGA chip to the CPCI chip. The other operation mode, packets shall be transferred from the CPCI chip to the CPU TX queue in the NetFPGA chip, using the pins of the CPCI chip and the NetFPGA chip.(Naous et al. 2008b)
- User Data Path (UDP): Includes blocks Input Arbiter, VLAN Remover, Watchdog, Output Port Lookup, VLAN Adder and Output Queue. This block function is to process and forward packets. It collects the packets from 8 inputs (4 CPU queue, and 4 DMA queue) (Moore et al. 2010) and transfers packets to 8 outputs respectively.
- NF2 MAC: This block communicates with MAC to Chip PHY to transmit and receive data through Ethernet ports.

### Detailed description of the input and output signals on the power manager

**Figure 6:**
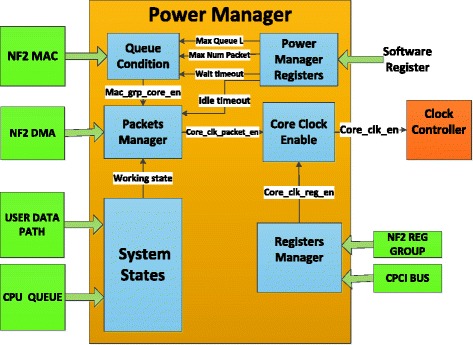
**The input and output signals on the Power Manager Block.**

According to the structure of the OpenFlow Switch based on NetFPGA platform, we design Power Manager Block including input and output signals that connect other blocks shown Figure 6.

Our design contains six small functional blocks: System States, Power Manager Register, Queue Condition, Packets Manager, Registers Manager and Core Clock Enable. The details of these blocks are designed as follows:

#### System states

This block is responsible for reporting a packet processing of the Openflow Switch. Inputs of this block are the signals from the User Data Path and the CPU Queue block. These inputs are synthetized to four signal groups that report operating states of inside blocks on the Openflow Switch (Table 1).

**Table 1:** **Four signal groups**

**Signal groups**	**Function**
work_udp_grp0 = udp_in_wr| vlan_remover_out_wr| (!opl_in_fifo_empty);	Report UDP is processing and forwarding packets.
work_udp_grp1 = vlan_adder_out_wr | udp_out_wr;	
work_cputx_grp = cpu_q_dma_wr_pkt_vld | cpu_q_dma_wr;	Report NF2 DMA is sending data to CPU DMA Queue.
work_cpurx_grp = cpu_q_dma_pkt_avail | cpu_q_dma_rd_rdy;	Report CPU Queue is receiving packets.

Based on signals from Table 1, the System States will report a signal - working_state to inform active states of the User Data Path and the CPU Queue block. If working_state = 1, the system is working and the switch still operates normally. If working_state = 0, packets have finished the packet processing and then we can reduce the operating frequency to 0 MHz to save energy.

#### Power manager registers

We can control the operation of switches through the Power Manager Registers block. This block is responsible for communicating with the registers to read and write the values which are configured by software. Some thresholds can be set and provided to the other blocks as:

- ➢ Max Queue Length is the maximum of queue length. When a queue length is equal to this threshold, a switch automatically changes to its normal operating status in order to forward packets.
- ➢ Max Packet Number is the largest number of packets on the input queue. When the number of packets at the input queue is equal to this threshold, the switch also changes to its normal operating state.
- ➢ Idle Timeout is the idle time of a switch after completing the packet processing. During this course, there is no traffic flowing through the switch and then it will sleep to save energy.
- ➢ Wait Timeout is a wait time threshold of packets in input queue. Wait time of a flow starts counting when a first packet received completely on the input queue. If the wait time is equal to the threshold - Wait timeout then the switch also changes to its normal operating state.

#### Queue condition

**Figure 7:**
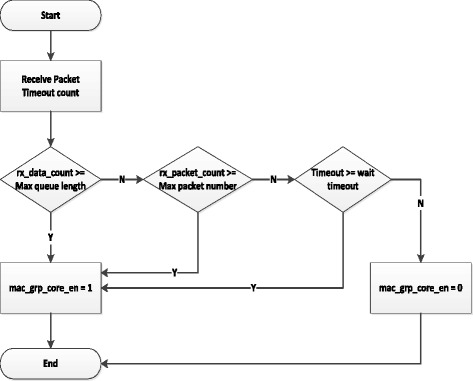
**Queue condition block working principle.**

Inputs of this block are the signals from NF2 MAC as: rx_data_count is the size of input queue; rx_packet_count is the number of packets on the input queue. Queue Condition block compares these signals with its thresholds that are output signals of the Power Manager Registers. If the value of rx_data_count is larger than that of the Max Queue Length, or the rx_packet_count is larger than the Max Packet Number or the wait time of a first packet is larger than the Wait Timeout, then this block will report an output signal – mac_grp_core_en to change the operating frequence of the Openflow Switch as the working principle shown in Figure 7.

#### Packets manager

This is a main block of the Power Manager. The function of this block is to manage the switch operating states. An input signal of this block named dma_vld_c2n reports the switch will receive packets via the DMA bus. Moreover, other inputs include the mac_grp_core_en that requires from the Queue Condition block, the working_state that informs active states of User Data Path and CPU Queue, and the Idle Timeout that is provided to Power Manager Registers.

From these signals, we define three new modes for the Openflow Switch as below:

- WORKING mode: This mode is the normal state of a switch. The operating frequence is provided at 125 MHz in order that the switch can receive and transmit data to other devices on the network.
- IDLE mode: This mode is activated when there is no traffic for processing. However, the operating frequence is still maintained at 125 MHz to wait next packets.
- SLEEP mode: This mode is activated when there is not any traffic going through the switch. After idle time of the switch equals to a threshold – idle timeout then the operating frequence shall be reduced to 0 MHz in order to save energy.

**Figure 8:**
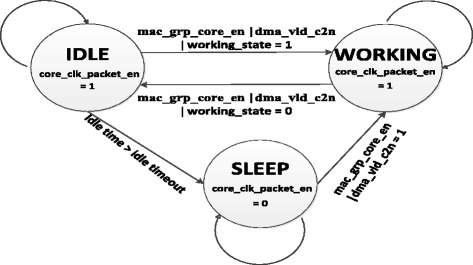
**States of Openflow Switch.**

The change of these modes is shown in Figure 8. At the WORKING mode and IDLE mode, a signal core_clk_packet_en = 1 requires the operating frequence to be maintained at 125 MHz; whereas the frequence is reduced to 0 MHz at the SLEEP mode in order to save consumed energy of the Openflow Switch.

#### Registers manager

This block is responsible for reporting the state of registers system on a switch. It receives signals from the registers such as the work_reg_grp signal reports queues of Nf2 Reg Group are processing and the cpci_bus_dv signal reports the switch will receive packets via the PCI bus. Either work_reg_grp = 1 or cpci_bus_dv = 1 Register Manager block will report an output signal – core_clk_reg_en to require the switch to operate normally at 125 MHz.

#### Core clock enable

**Figure 9:**
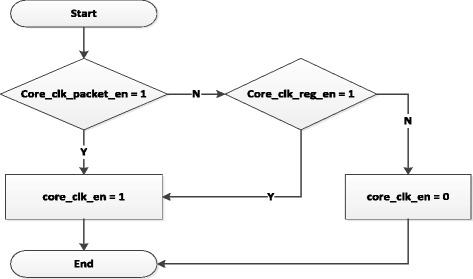
**Core Clock Enable block working principle.**

Based on signals of the Packets Manager and the Register Manager, Core Clock Enable block will report a signal - core_clk_en to change the operating frequence of the Openflow Switch as the working principle shown in Figure 9.

## Design of clock controller block for the openflow switch

**Figure 10:**
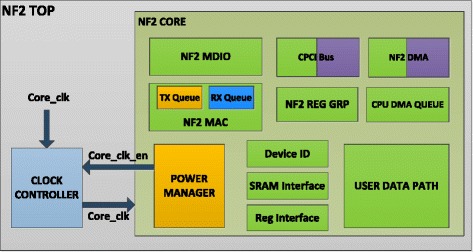
**Core_clk is provided to the inside blocks of the Openflow Switch.**
- Grx_clk (125 MHz): Clock used to receive data to four Ethernet ports. The blue block – RX Queue in Figure 10 uses this clock.
- Cpci_clk (62.5 MHz): Clock used for communication blocks between NetFPGA and PC through the PCI Bus. The violet blocks in Figure 10 use this clock.
- Core_clk: The main clock used for mosts of the functional blocks of the Openflow Switch. The frequence of this clock is 125 MHz to guarantee that the bandwidth on each Ethernet port is 1Gbps. In this paper, we intently impact on this clock to save energy.

The system of clock sources on an Openflow switch (OpenFlow Switch Specification Version 1.1.0 Implemented (Wire Protocol 0x02)) (Gibb et al. 2008) have four clock signals:

- Gtx_clk (125 MHz): Clock used to transmit data to four Ethernet ports. The orange blocks in Figure 10 use this clock.

**Figure 11:**
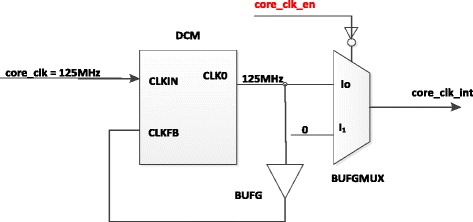
**Describe the Clock Controller.**

In this section, we have implemented a controlling module named *Clock Controller* that is responsible for changing the operating frequency of switch at 125 MHz or 0 in order to reduce the consumed energy. Clock Controller block is designed as shown in Figure 11 that includes Digital Clock Managers (DCM), BUFGMUX block and BUFG block. (“Virtex-II Pro and Virtex-II Pro X FPGA User Guide”, UG012 (v4.2) 5 November 2007)

.

DCM provides a wide range of powerful clock management features: Clock De-Skew, Frequency Synthesis, Phase Shifting and General Control Signals. The DCM contains a delay-locked loop (DLL) that can completely eliminate clock distribution delays, hence deskewing the DCM’s output clocks with respect to the input clock. The DLL contains delay elements (individual small buffers) and control logic. The control logic contains a phase detector and a delay line selector. The phase detector compares the incoming clock signal (CLKIN) against a feedback input (CLKFB) and steers the delay line selector, essentially adding delay to the output of DCM until the CLKIN and CLKFB coincide.

BUFGMUX can switch between two unrelated, even asynchronous clocks. With the output signal of Power Manager - core_clk_en, BUFGMUX selects operating frequencies such as 0 MHz or 125 MHz of core_clk for blocks inside a switch. Basically, a High on core_clk_en selects the I_0_ input (core_clk_in = 125 MHz), and a Low on core_clk_en selects the I_1_ input (core_clk_in = 0 MHz).

BUFG is a global clock buffer with one clock input (CLK125) and one clock output (CLKFB), driving a low skew clock distribution network.

When the switch is running at the sleep mode, Clock Controller does not provide frequence - core_clk for the inside blocks, which are the green blocks shown in Figure 10. Therefore, these blocks will not operate and sleep to save the consumed energy.

In our method, we can reduce the operating frequence to 0 MHz because we always maintain the performance of Power Manager and Clock Controller. The clock of these blocks is Gtx_clk. This is effective in saving consumed energy of the Openflow Switch.

## Experimental results

### Design test-bed system

**Figure 12:**
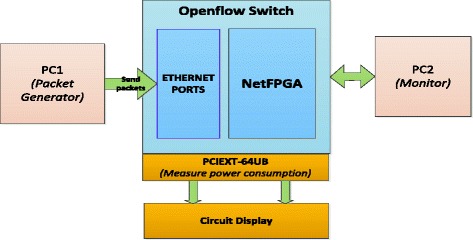
**Testbed system for saving energy Openfow Switch.**

In order to test and evaluate our design, we have built a hardware test-bed including an OpenFlow Switch based on NetFPGA-1G board (Figure 12). An OpenFlow switch version 1.0.0.4 (OpenFlow Switch Specification Version 1.1.0 Implemented (Wire Protocol 0x02)) based on NetFPGA version 3.0.1(Gibb et al. 2008) which is developed by Stanford University is used.

We also use PC1 to generate packets on links with different throughputs. PC2 is connected to the Openflow Switch and receives packets to PC1.

Power Measure Board and Circuit Display are used to read ADC value at test-points 3.3 V and 5.0 V via PCIEXT-64UB (Product Specifications and User Manual of PCI-EXT64U/UB and PCIeEXT-16HOT) and then calculate and display the consumed energy of the Openflow Switch.

### Define two modes for balance switch

We can propose and set many different modes for different requirements by changing thresholds such as the Idle Timeout, Max Queue Length, Max Number packet and Wait Timeout. In our experiment, we define two modes for the Balance Switch that is Low Power and Save Power as below:

#### Low Power mode

The purpose of this mode is to wake up the switch after receiving completely a packet on the input queue. We set thresholds includes: Idle Timeout = 5 clocks = 40 ns, Max Queue Length = 2000 bytes, Max Number packet = 1 packet, Wait Timeout = 12500 clocks = 100us.

#### Save Power mode

The purpose of this mode is to set parameters as the Idle Timeout, Max Queue Length, Max Number packet and Wait Timeout with the largest thresholds in order to save the largest power. We set these thresholds: Idle Timeout = 5 clocks = 40 ns, Max Queue Length = 5120 bytes, Max Number packet = 127 packets and Wait Timeout = 12500000 clocks = 100 ms.

### Measure consumed power of balance switch depending on throughput

**Figure 13:**
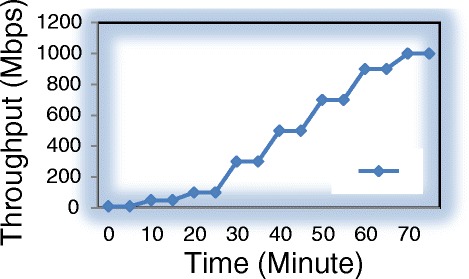
**The different levels of throughput.**

In our experiment, firstly, we generate packets with the different levels of input traffic from 0 to 1Gbps. (Figure 13). After that we measure the average power consumption of two cases: Normal switch, which does not integrate the saving energy function and Balance switch, which is our design at two modes: Low Power and Save Power. Measurement results are shown in Table 2.

**Table 2:** **Consumed power of normal switch and balance switch**

**Throughput (Mbps)**	**0**	**10**	**50**	**100**	**300**	**500**	**700**	**900**	**1000**
**Normal Switch (W)**	10.92	10.92	10.92	10.92	10.92	10.92	10.92	10.92	10.92
**Low Power mode (W)**	7.194	7.333	7.417	7.567	7.678	7.728	7.861	7.9	8.056
**Save Power mode (W)**	7.111	7.278	7.333	7.5	7.583	7.639	7.722	7.806	7.889

**Figure 14:**
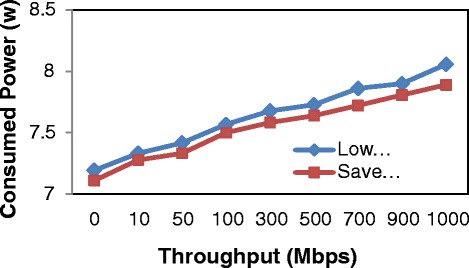
**Consumed power of normal switch and balance switch.**

According to the results in Table 2, we obviously realize that consumed power at Normal switch remained constant around 11 W although throughput varies. Moreover, we can see as shown in Figure 14, it is clear that the power consumption of Balance Switch increases between 0 Mbps and 1000 Mbps. Overall, the consumed power of Low Power mode is larger than that of Save Power mode. The figures for the former rises from 7.2 W to 8.06 W, with saving power around 26.2% to 34.0% and the figures for the latter also increases from 7.1 W to 7.9 W, with saving power about from 27.7% to 35.0%. In general, the largest saving power is at the Save Power mode, accounting for about 35.0%.

### Measure consumed energy of balance switch with different input traffic

**Figure 15:**
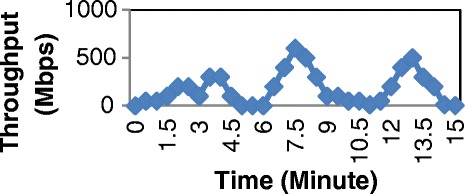
**Profile of input traffic 1.**

**Figure 16:**
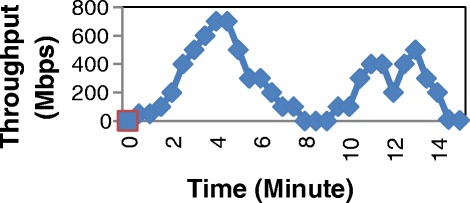
**Profile of input traffic 2.**

**Figure 17:**
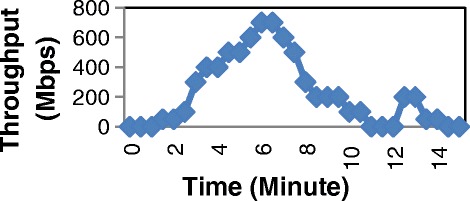
**Profile of input traffic 3.**

In the second stage of the test, we generate different input profiles on PC1 (Figure 15, Figures 16 and Figure 17). Then we also measure the total consumed energy of the switch within approximately 15 minutes for each profile with two cases: Normal switch and Balance switch at two modes: Low Power and Save Power. The results are shown in Table 3.

**Table 3:** **Consumed and saved energy of balance switch**

**Profile**	**Consumed energy (J/15 min)**			**Saved energy (%)**	
	***Normal Switch***	***Low Power***	***Save Power***	***Low Power***	***Save Power***
**Profile 1**	10314 J	7088 J	6881 J	31.28%	33.28%
**Profile 2**	10314 J	6705 J	6528 J	35.0%	36.7%
**Profile 3**	10314 J	6852 J	6695 J	33.57%	35.1%

As shown in Table 3, you can see that the saved energy of switch depends on the input flow. In experiment, the saved energy of Save Power mode is larger than that of Low Power mode. There is around from 30% to 35% energy saved in comparison with Normal switch. In (Thanh et al. 2012) (Heller) shown that the traffic through switches and routers varies according to time the input traffic reaches the peak during the day and falls at night. Therefore, the input traffic has lower level within one day (from 0 hour to 6 hour everyday), energy is saved considerably.

## Evaluation testbed system

### Evaluate processing time of our method

Before delving into the details, let’s have the following notations: T_a_ (k) is arrival time of packet k. Arrival time of a packet is the time at which the last bit of the packet has arrived in the input queue (RX Queue). T_d_ (k) Departed time of packet k. Departed time of a packet is the time at which the last bit of the packet has arrived in the output queue (TX Queue. L (k) is packet length of packet k. C is link rate (the same for output and input). We have processing time of the packet k on our system is:

3 $$ {\mathrm{T}}_{\mathrm{process}}\left(\mathrm{k}\right) = {\mathrm{T}}_{\mathrm{d}}\left(\mathrm{k}\right)\ \hbox{--}\ {\mathrm{T}}_{\mathrm{a}}\left(\mathrm{k}\right) $$

Ethernet devices must allow a minimum idle period between transmissions of Ethernet packets known as the **interpacket gap** (IPG). With the packet k, interpacket gap (IPG) is the gap time from a first bit of the packet k + 1 already arrived in the input queue to a last bit of the packet k already arrived in the output queue (TX Queue). Therefore, the interpacket gap between packet k and k + 1, denoted by T_IPG_ (k) is calculated as below:

4 $$ {\mathrm{T}}_{\mathrm{IP}G}\left(\mathrm{k}\right) = {\mathrm{T}}_a\left(\mathrm{k}+1\right) - \mathrm{L}\ \left(\mathrm{k}+1\right)/\mathrm{C}\ \hbox{--}\ {\mathrm{T}}_{\mathrm{a}}\left(\mathrm{k}\right) $$

If interpacket gap T_IPG_ (k) > T_process_ (k) + Idle Timeout then our system will sleep to save energy. Normally, (T_process_ (k) + Idle Timeout) is about μs that is effective in saving energy.

### Evaluate QoS for balance switch

#### Delay time of a packet

- ➢ Low Power

In this mode, we configure Max Packet Number = 1, instance, when the switch receives completely a packet in the input queue, the system will turn on and operate normally. Therefore, the switch that operates at Low Power mode has latency equal to the wake-up time about 3–4 clock cycles, which is approximately 24 ns - 32 ns. On the other hand, in one frequence, the general openflow switch will receive 8 bits (1byte) into queues. If a packet has average size about 1000 bytes; the received time is about 1000 clock cycles. As you can see, the delay time caused by our method is negligible in comparison with the wait time in queues.

- ➢ Save Power

In this mode, we set parameters with the largest thresholds as Idle Timeout = 5 clocks = 40 ns, Max Queue Length: 5120 bytes, Max Number packet: 127 packets and Wait Timeout = 12500000 clocks = 100 ms. Thus, latency depends on the distribution and the Inter-departure time of packets in the network. We can evalute the maximum delay time in our method that is calculated as below:

5 $$ {\mathrm{T}}_{\max\ \mathrm{delay}} = \mathrm{Idle}\ \mathrm{timeout} + {\mathrm{T}}_{\mathrm{process}} $$

In this case, we can reduce the idle timeout to proper the quality of service.

#### Packet dropping probability

In our switch, we control operating modes of switches based on queue size, when queue length equals the threshold –Max Queue Lengh then the switch starts in processing and transmiting packets to receivers. Besides, the value of Max Queue Lengh at the Low Power mode and Save Power mode is set at 2000 bytes and 5120 bytes, respectively. While size of an input queue (RX Queue) is 8096 bytes that is larger than the Max Queue Lengh. On the other hand, in (Naous et al. 2008a) presents implementation of the Openflow Switch can hold more than 32,000 exact-match flow entries and is capable of running at line-rate across the four NetFPGA ports. Thus, when the switch is turned on, it runs at line-rate. Our method also does not drop packets compared with the normal switch.

## Conclusion

In this paper, we have designed and implemented ***Balance Switch*** to reduce power consumption. We have proposed two modes: ***Low Power*** and ***Save Power*** for the ***Balance Switch***. Additionally, we have also built the test-bed system and have precisely measured the energy of the whole switch saving of about ***30-35%.*** As a result, the saved energy at the ***Save Power*** is larger than the ***Low Power*** mode. Besides, we have also assessed and shown that our switch do not drop packets compared with the normal Openflow Switch. The results present in this paper can be readily used in reducing energy consumption on data centers. Moreover, the results also contribute to develop on green networking.

In the future, however, we will design other test-bed to study and propose some thresholds such as Idle Timeout, Max Queue Length, Max Number packet and Wait Timeout for different services. Besides, we will also combine with previous methods in (Vu et al. 2014) to measure and evaluate power saving for a large network.
